# The multimorbidity interaction severity index (MISI): A proof of concept study

**DOI:** 10.1097/MD.0000000000006144

**Published:** 2017-02-24

**Authors:** Dimitri Gassmann, Marcus Cheetham, Klarissa Siebenhuener, Barbara M. Holzer, Claudine Meindl-Fridez, Florian F. Hildenbrand, Vanessa Virgini, Mike Martin, Edouard Battegay

**Affiliations:** a Department of Internal Medicine, University Hospital Zurich; b Center of Competence Multimorbidity; c University Research Priority Program “Dynamics of Healthy Aging,” University of Zurich; d Department of Dermatology, University Hospital Zurich; e Division of Gerontopsychology and Gerontology, Department of Psychology, University of Zurich, Zurich, Switzerland.

**Keywords:** computerized decision support, drug–disease interactions, drug–drug interactions, MISI, multimorbidity, severity index, therapeutic conflict

## Abstract

Therapeutic decision-making for patients with multimorbidity (MM) is challenging. Clinical practice guidelines inadequately address harmful interactions and resulting therapeutic conflicts within and among diseases. A patient-specific measure of MM severity that takes account of this conflict is needed.

As a proof of concept, we evaluated whether the new Multimorbidity Interaction Severity Index (MISI) could be used to reliably differentiate patients in terms of lower versus higher potential for harmful interactions.

Two hypothetical patient cases were generated, each with 6 concurrent morbidities. One case had a low (i.e., low conflict case) and the other a high (i.e., high conflict case) potential for harmful interactions. All possible interactions between conditions and treatments were extracted from each case's record into a multimorbidity interaction matrix. Experienced general internists (N = 18) judged each interaction in the matrix in terms of likely resource utilization needed to manage the interaction. Based on these judgements, a composite index of MM interaction severity, that is, the MISI, was generated for each physician and case.

The difference between each physician's MISI score for the 2 cases (MISI_*diff*_) was computed. Based on MISI_*diff*_, the high conflict case was judged to be of significantly greater MM severity than was the low conflict case. The positive values of the inter-quartile range, a measure of variation (or disagreement) between the 2 cases, indicated general consistency of individual physicians in judging MM severity.

The data indicate that the MISI can be used to reliably differentiate hypothetical multimorbid patients in terms of lesser versus greater severity of potentially harmful interactive effects. On this basis, the MISI will be further developed for use in patient-specific assessment and management of MM. The clinical relevance of the MISI as an alternative approach to defining MM severity is discussed.

## 1 Introduction

Multimorbidity (MM) refers to the presence of multiple concurrent acute or chronic diseases within a person.^[1,2]^ Therapeutic decision making for multimorbid patients is challenging.^[3–6]^ This is because decisions rely on recommendations from clinical practice guidelines that were developed for the treatment of single diseases.^[7–9]^ With some exceptions,^[9–12]^ these guidelines are based on evidence from studies that excluded or under-represented multimorbid cases.^[13–16]^ This mono-morbid approach to therapeutic decision-making in MM does not, therefore, adequately address the combined impact of potentially harmful disease–disease, drug–disease, and drug–drug interactions (DDIs) and multiple drug regimens^[9,17–21]^ or adequately guide the clinical decision-maker through the therapeutic conflict.

Therapeutic decision making in MM requires consideration of potential DDIs^[22,23]^ and their combined impact to determine a suitable clinical strategy, on a case-by-case basis. In some patients, co-occurring conditions and treatments can be managed without risk of harmful effects (e.g., physical exercise for hypertension, diabetes, and dyslipidemia) due to the treatment of one of the constituent conditions. But therapeutic conflict of various degrees of severity is typically encountered in that the treatment for 1 condition is contraindicated by the presence of 1 or more other conditions or treatments.^[24]^ This applies, for instance, to concurrent gastrointestinal bleeding and anticoagulant treatment for heart disease, concurrent severe lung disease and benzodiazepine treatment for a sleeping disorder, or severe lung disease and opiate treatment for a pain disorder.^[8,25]^ The overall complexity of such cases can place particularly high demands on the clinical decision-maker in reconciling the range of harmful interactions with a therapeutic strategy that is specifically tailored to the particular needs of the patient.^[26,27]^

Intensive work to support clinical decision making in MM is in progress (e.g.,^[9,11,13,28]^). But, at present, there is no instrument for measuring patient-specific burden of harmful DDIs that can be applied to any combination of medical conditions. A reliable measure of MM burden would facilitate the development of tools and guidance to support diagnostic and therapeutic decision making and provide a valuable frame of reference for comparing research findings (e.g.,^[29]^). The most commonly applied measures in the field of MM were originally constructed to support clinical decision making in specific patient groups in hospital settings or for research purposes (e.g.,^[30–34]^). These measures typically quantify the degree of MM in an individual by summing the number of concurrent diseases. Given the limitations of this approach, these measures have been superseded in part by the use of indices. Indices use weights to differentially assess each disease or condition, for instance, in terms of the physician's judgement of likely severity or resource utilization^[35–39]^ or prognosis.^[40]^ While this approach is considered useful for informing the planning and prioritization of treatment,^[41]^ a complex case can render assessment particularly difficult.

**Figure 1:**
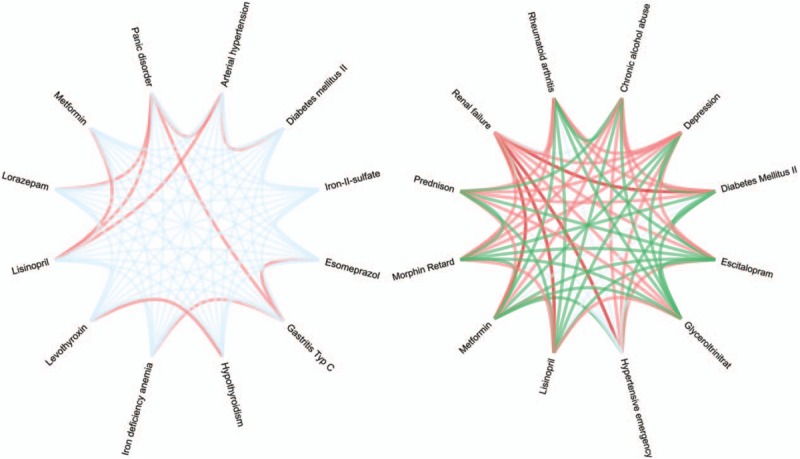
Network graph generated from the multimorbidity interaction matrix on the basis of the participants’ ratings of interaction severity, showing (A) the graph for the low conflict case with little risk of harmful interactions, and (B) the graph for the high conflict case with a high risk of harmful interactions.

We developed therefore a web-based decision support tool for use at patient encounter to facilitate assessment of complex cases and therapeutic decision making. The tool has 3 main features. First, it generates a case-specific multimorbidity interaction matrix of all potential DDIs from the patient record. The matrix is used to score each DDI in terms of likely resource utilization (i.e., expected intensity of effort needed to manage the interaction) and is intended to aid the physician's consideration of harmful DDIs and points of caution, uncertainty, and priority in the clinical management of the specific case. Second, the tool generates a network graph from these scores to help the physician visualize his or her assessment of the case (see Fig. 1). Third, the tool generates an overall composite score, or multimorbidity interaction severity index (MISI), of the case as a summary evaluation of the interaction severity of the case on a comparative scale. This preliminary study used the MISI as a basis for the initial evaluation of the tool. We tested the expectation that the MISI could be used to reliably differentiate 2 hypothetical multimorbid patient cases in terms of potential lower versus higher therapeutic conflict. Each case comprised a cluster of 6 similar concurrent conditions, in 1 case with a low and in the other a high potential for harmful interactions and therapeutic conflict.

## 2 Methods

### 2.1 Participants

Eighteen senior physicians (8 females, mean years of clinical experience as General Internists = 12.94, *SD* = 6) volunteered to participate. All participants were senior General Internists and staff members of the Department of Internal Medicine (General Internal Medicine), University Hospital of Zurich, Switzerland and native or fluent speakers of Swiss German (or Standard German) and fluent speakers of English. This pilot study was designed to explore the new index of MM severity on the basis of different patterns of responses of the participants in judging the severity of patients DDIs in terms of expected resource utilization. In the absence of comparable data for the application of this construct to all combinations of potential DDIs, the sample size was selected on the basis of the experience gained developing the MISI. Sample size calculations for future development of the MISI can be based on estimates derived from this study. Local ethics committee approval was not required for the purpose of our sample of physicians evaluating hypothetical patients. Written informed consent was obtained according to the guidelines of the Declaration of Helsinki, data coded anonymously, and each physician debriefed at the end of the study. The data used and analyzed for the current study are available from the corresponding author on reasonable request.

### 2.2 Materials

#### 2.2.1 Hypothetical patients

Authors EB and DG generated 2 hypothetical clinically plausible patient cases, a low and a high conflict case, each with 6 concurrent morbidities. In the low conflict case, the selection of concurrent conditions and treatments was deemed to have a low risk of harmful interactive effects and therapeutic conflict, whereas in the high conflict case these were considered to carry a high risk of harmful interactive effects and therapeutic conflict (see Table 1). The data of diagnosed conditions and medications of the hypothetical cases were imported into the web-based MISI.

**Table 1:**
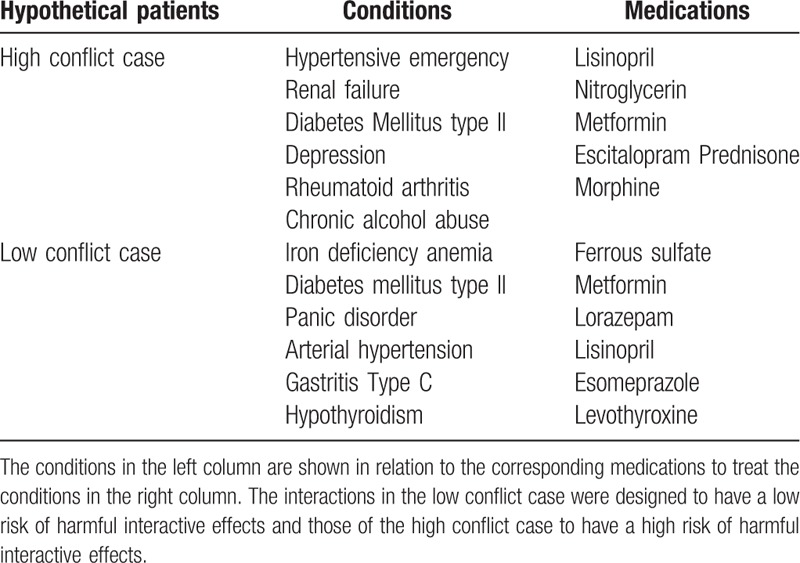
Conditions and medications of 2 hypothetical patient cases.

#### 2.2.2 Technical description of the web-based MISI

The MISI is integrated into a web-based decision support tool. The tool comprises a platform-independent, web browser-based system, with a graphical user interface and a dedicated server component. It is built on open source technology, including node.js and MongoDB for server-side application (express.js, passport.js, mongoose.js, socket.io) and HTML, CSS, and various JavaScript libraries (angular.js, d3.js, and Twitter Bootstrap) for the browser client.

### 2.3 Study procedure

All participants were tested individually in a small quiet room, located at the University Hospital of Zurich, and were blind to the design of the hypothetical cases. The experiment lasted approximately 35 min. The 2 cases were presented across participants in counter-balanced order. The experimental setup included a 13-inch MacBook Pro with a Retina display and an optical mouse. The server of the web-based tool operated on the machine locally, with the webapp displayed in Firefox v42.0 in full-screen mode. Participants provided demographic information (e.g., age, clinical experience as General Internists). The web-based MISI was then presented to the participants. It was explained how to use the MISI and ensured that the explanation had been understood.

Each participant subsequently read the written instructions presented on the PC monitor as to how to proceed. First, and using the web-based MISI, a panel displayed information on the hypothetical case (e.g., the reason for the encounter, relevant findings in clinical examination, further relevant findings in diagnostic tests, medical history). Second, the hypothetical list of conditions and medications was displayed and the participants were required to rate each condition as either active or inactive and each medication as either modified or unmodified. The web-based MISI provides a definition of active and modified: Active is any medical condition that requires current and ongoing diagnostic or therapeutic (pharmacological or other) attention or measures, and modified is any medication or other therapeutic measure that needs to be altered in content or dose. These and all further ratings were conducted by mouse click on the checkboxes of the MISI's interface. Third, upon completing the last step, the MISI automatically generated a panel displaying a multimorbidity interaction matrix of all possible interaction pairs extracted from the list of active conditions and medications. Participants were required to rate each interaction pair on a qualitative 4-point severity scale of resource utilization (i.e., expected intensity of effort needed to manage the interaction). The scale ranges from “not harmful” (requiring therefore no action) to “life threatening” (requiring therefore severe action), as shown in Table 2. The severity scale also included a further option “don’t know” to indicate that the participant is unable to judge whether a potential interaction might be harmful.

**Table 2:**
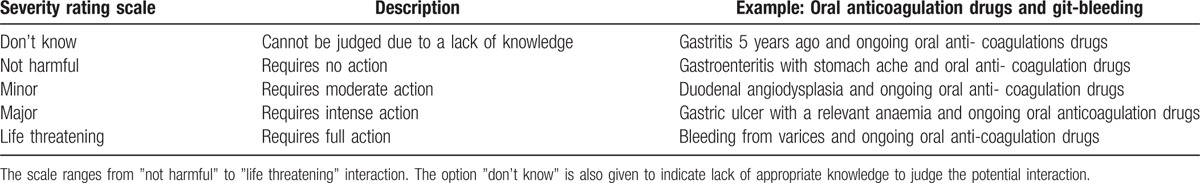
The table shows the 4-point severity rating scale, with a corresponding description and example (condition and medications) for each level of severity.

Finally, after completing the multimorbidity interaction matrix, the composite MM severity score was computed automatically and displayed above the matrix. The composite score of the MISI is computed by summing up the total number of conditions, medications, active conditions, modified medications, and the number of all interactions rated in terms of severity as minor, as major (multiplied by 3), and as life threatening (multiplied by 10) (see Table 3). Upon completion of the test, the participant was debriefed and asked to report any difficulties experienced using the web-based MISI.

**Table 3:**
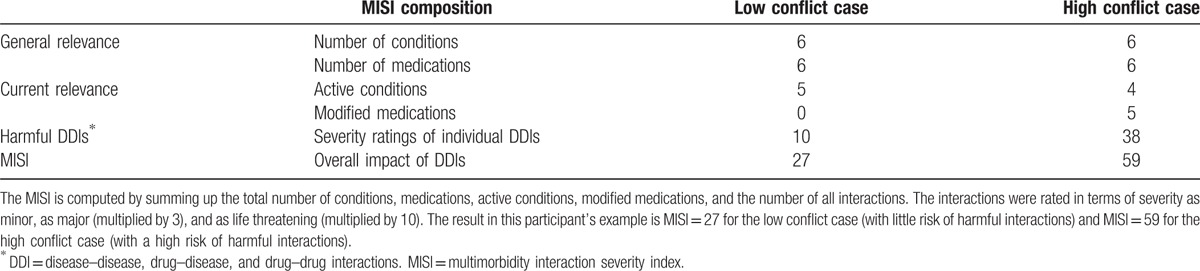
The table shows an example of the composition of the MISI on the basis of one of the participant's ratings of a low and a high conflict case.

To aid decision-making, the web-based decision support tool also generates a visually intuitive network graph to represent the internist's judgements of interaction severity, as rated in the multimorbidity interaction matrix (see Fig. 1). The graph provides a visual impression of the overall MM severity of the case. The aim of this visualization is to support the physician by highlighting nontrivial relationships that might otherwise be overseen in the complex patient by showing the relevant interactions between the conditions and treatments weighted according to the internist's judgements of severity. MM severity of each case can be visualized in network graph generated from the multimorbidity interaction matrix (see Fig. 1).

## 3 Results

All data analyses were performed using SPSS version 21.0 (SPSS Inc, Chicago, IL). There were no missing data.

One might anticipate that the high conflict case demands more careful consideration in making judgements about potential harmful interactions and resource allocation than does the low conflict case and that greater consideration is reflected in the time taken to judge each case. In fact, the mean time taken by the internists to score the interactions in the MISI's multimorbidity interaction matrix was *M* = 11.58 min (*SD* = 4.21; range = 3.25–21.48) for the low conflict case and *M* = 15.76 min (*SD* = 4.99; range = 5.27–24.37) for the high conflict case. A paired *t* test showed that the high conflict case took significantly longer to score than the low conflict case, *t*_(17)_= 4.53, *P* < 0.001.

Each patient case was designed to have 6 generally relevant medical conditions and medications. Before using the multimorbidity interaction matrix to rate each potential DDI in terms of likely resource allocation, the internists were first required to judge whether each condition was currently active and whether the medication used needed to be modified. Before testing for differences between the 2 hypothetical cases, the Kolmogorov–Smirnov test for normality was conducted.^[42]^ This indicated that the distribution of active condition judgements deviated significantly from a normal distribution in the low (*D* = .298, *P* < 0.01) and high conflict case (*D* = .274, *P* < 0.01). A Wilcoxon Signed-ranks test was therefore applied,^[43]^ this showing no significant difference between the high (*Mdn* = 5) and low conflict case (*Mdn* = 6) in terms of active judgements, *Z* = −0.78, *p* = 0.44. The Kolmogorov–Smirnov test for normality indicated that the distribution of modified medication judgements also deviated significantly from a normal distribution for the low (*D* = 0.226, *P* = .02) but not for the high conflict case (*D* = 0.177, *P* *=* 0.14). A Wilcoxon Signed-ranks test was therefore performed. This showed that the high conflict case (*Mdn* = 3.5) was judged as requiring modification significantly more often than the low conflict case (*Mdn* = 1), *Z* = −2.37, *P* = 0.018.

In other words, the preceding analyses show that the internists judged no difference between the 2 cases in terms of the number of active concurrent conditions. But they did judge a difference in terms of the number of required medication modifications. Given the same number of conditions in each case, the results suggest that the cumulative number of concurrent conditions or conditions (i.e., the oft used definition of MM) does not necessarily reflect the clinical resource effort needed to manage (i.e., in this case, to modify medications) in a multimorbid patient.

We then evaluated whether the MISI could be used to reliably differentiate between the 2 patient cases in terms of lower versus higher therapeutic conflict. To do this, the difference between each physician's composite MM severity score, MISI_*diff*_, was computed and used for further analyses.

The descriptive data for MISI_*diff*_ largely show positive values (*Min* = -5, *1*^*st*^*Quartile* = 16.75, *Mdn* = 32.50, *M* = 30.44, *3*^*rd*^*Quartile* = 47.75, *Max* = 64.00). This means that most raters gave the low conflict case a lower score than the high conflict case. In fact, the mean 50% of internists produced a MISI_*diff*_ value between 16.75 and 47.75 between the 2 cases. The positive values of the inter-quartile-range (IQR = 31) and the standard deviation (*SD* = 19.84) for MISI_*diff*_, used as measures of variation (or disagreement) between the 2 cases, suggest overall consistency of the individual physicians in their judgments. However, the minimum value of −5 indicates that at least 1 physician rated the low conflict case as having slightly greater MM severity than the high conflict case.

**Figure 2:**
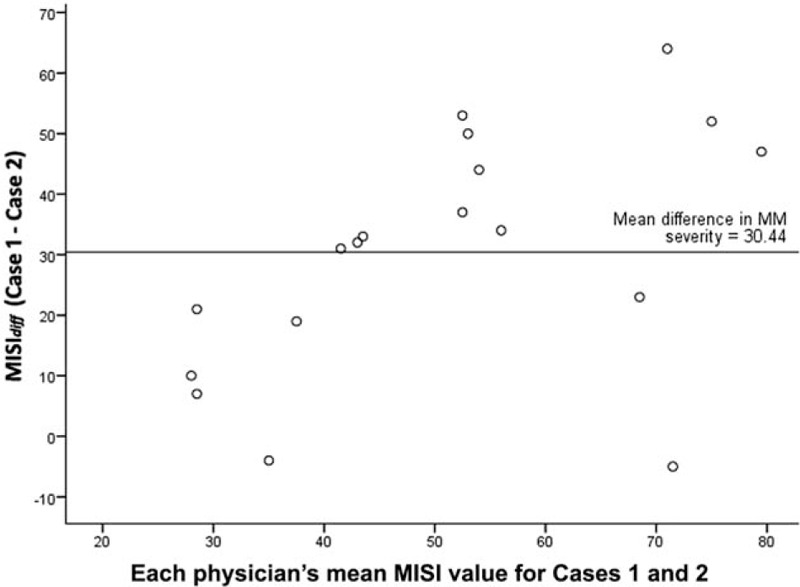
The scatter plot (based on the Bland–Altman approach) depicts the mean of a particular physician's 2 MM severity scores for cases 1 and 2 on the X-axis and the difference between that physician's 2 MM severity scores for cases 1 and 2 (i.e., high conflict case minus the low conflict case, or MISI_diff_) on the Y-axis. The mean overall mean difference between cases 1 and 2 across all physicians is *M* = 30.44 (SD = 19.83).

For illustrative purposes, MISI_*diff*_ can be shown in a scatter plot based on the Bland–Altman (B&A) approach^[43]^ (see Fig. 2). The scatter plot allows the pattern of MISI data to be inspected: each point in the plot shows the mean of each physician's MM severity scores for cases 1 and 2 on the X-axis and each physician's MISI_*diff*_ for cases 1 and 2 on the Y-axis. The overall mean of MISI_*diff*_ across all physicians is *M* = 30.44 (*SD* = 19.83). Closer inspection of Fig. 2 reveals that 2 of the 18 physicians show differences in their MM severity scores of close to zero, meaning that these 2 judged the MM severity of the 2 cases as being highly similar.

The Kolmogorov–Smirnov test for normality indicated that the MISI_*diff*_ distribution did not deviate significantly from a normal distribution (*D* = 0.122, *P* = 0.200). A one sample *t* test was conducted to test the null-hypothesis that MISI_*diff*_ is zero, that is, the physicians judged the 2 cases similarly in MM severity. This test showed that the difference between the MISI_*diff*_ values (*M* = 30.44, *SD* = 19.83) and zero was highly significant, *t*_(17)_ = 6.51, *P* < 0.001, 95% CI 20.58, 40.31. This indicates that the physicians distinguished between each case in terms of lower and higher MM severity and therapeutic conflict.

## 4 Discussion

As a proof of concept, the present study evaluated whether the MISI could be used to reliably distinguish between 2 clinically relevant but hypothetical patient cases in terms of the intensity of effort (i.e., resource utilization) required to clinically manage harmful interactions. The 2 patients were designed to have either a low or high risk of harmful interactive effects with a corresponding degree of therapeutic conflict (see Table 1). In the high conflict case, the design included hypertensive emergency, renal failure, and diabetes mellitus type II as a typical cluster of conditions in MM.^[44]^ Depression, often associated with noncompliance, may cooccur with these conditions, and noncompliance may be associated with hypertensive emergencies.^[44–46]^ Diseases such as rheumatoid arthritis can cooccur with the other conditions, though not necessarily in relation to the typical cluster.^[47]^ In the low conflict case, these prevalent diseases (iron deficiency anemia, diabetes mellitus type II, panic disorder, arterial hypertension, gastritis type C) often cooccur, with the exception of hypothyroidism, but not as a typical MM cluster.^[48–50]^ Consistent with their design, the analyses showed that the low conflict case was judged to be of significantly less MM severity than the high conflict case. The data thus support the use of the MISI as a means to distinguishing between different patient cases in terms of the severity of harmful interactions and therapeutic conflict.

This web-based tool is novel both as an instrument and for physicians who have never used a multimorbidity severity matrix to judge harmful interactions, as in our case. In this sense, the physicians were also under investigation. As a precursor to a conventional inter-rater agreement analysis, in which we would have a larger range of hypothetical or real cases (e.g.,^[51]^), this preliminary study was designed to give insight into the pattern of MISI data generated by the physicians while applying careful experimental control to the 2 cases. The general difference in the pattern of MISI data between the cases suggests that the physicians similarly understood and used the multimorbidity severity matrix to judge expected resource utilization. While this suggestion is supported by the physicians’ feedback at debriefing, we are mindful of potential sources of error in our new approach. We consider therefore the present study in terms of a number of factors that require more attention before conducting a conventional study of inter-rater agreement.

This tool is intended for use as a brief instrument to facilitate assessment of complex cases and illness severity. The time taken for our experienced general internists (who received only a brief introduction to the tool) to complete their task averaged 12 minutes for the low conflict case and 16 minutes for the high conflict case. This difference likely reflects a greater level of difficulty in assessing the high conflict case, but there was large variability between internists. This ranged from 3 to 24 minutes. This variability might suggest potential for improving the time to administer the instrument. Multiple factors, including uncertainties in MM and differences in internists’ knowledge and experience, influence the process of evaluating the complex multimorbid patient and this is likely to reveal differences between clinical decision makers^[52,53]^ in terms of completion time and the absolute values of the MISI.^[54–56]^

While the internists were all highly experienced in MM, they were not trained in or otherwise accustomed to using a multimorbidity interaction matrix to score harmful interactions. Training would help to ensure a common understanding of the matrix and its scoring procedure and enhance agreement between the physicians.^[57,58]^ To develop a common understanding (referred to in rater training as a frame of reference, e.g.,^[59]^) potential sources of disagreement between users of this tool need to be identified.^[60]^ A primary consideration in the present study is the extent to which the descriptors used to characterize the severity scale (see Table 2) are open to discrepant interpretation by different internists. Severity was operationally defined in terms of internists’ subjective judgements of projected resource utilization (i.e., intensity of effort) to manage harmful interactions. At debriefing, this definition was not reported as presenting any difficulties, and the inter-quartile-range, used as a measure of variation (or disagreement) between the 2 cases, suggests general consistency (albeit with the exception of 3 internists) in the use of the severity scales across the 2 cases (cf.^[61]^).

It is possible that good consistency (or, in a study with more than 2 patient cases, good interrater agreement) within a single-center sample reflects a good degree of shared knowledge of and routine in applying available center-specific resource and care management procedures. Any differences across centers in resources and procedures for MM in general or for specific clusters of MM might have a different influence on physicians’ judgements of resource utilization for the treatment of MM patients. This might result therefore in some degree of systematic variation in inter-rater agreement across centers. As a broadly applicable measure, the MISI might provide a means to evaluating this possibility. But the present study revealed that 2 of the physicians judged the low conflict case as slightly more severe than the high conflict case. Besides training, knowledge, and experience, testing is needed to establish what other (e.g., motivational) factors might influence the use of the tool for judging MM severity.

These factors might include experimental considerations. Every effort was made during recruitment and at instruction to ensure that participants were not aware of any potential difference in the severity of the hypothetical cases. Once physicians began using the multimorbidity severity matrix to rate the expected resource utilization of potentially harmful interactions, the physician may have tried to infer from those ratings the general severity of the case. The motivation behind using a multimorbidity severity matrix is that it might make it easier to build up a picture of a more complex case. This might be particularly helpful for less experienced physicians. But from an experimental perspective, we cannot exclude the possibility that a physician's ratings influenced subsequent ratings in the matrix, or that the severity ratings were biased by the physician's anticipation of the general severity of the case before completing the matrix. While we ensured that conditions and medications were always presented in the same sequence across all participants, future experiments could randomize the order of entries in the matrix to test for such bias. On the other hand, the network graph and the final index were only accessible to the physician after the completion of the matrix and did not influence the physician's ratings.

The strengths and weaknesses of the web-based tool may be considered in relation to its main features. The multimorbidity interaction matrix may help the physician to build up a cohesive and systematic assessment of potentially harmful DDIs. This can be important because, unlike the well-structured reporting of diagnoses and conditions, interactions are often described, sometimes lengthily, in free text. Given the potential complexity of many cases, the matrix might be especially helpful for less experienced physicians. To ensure the relevance of the judgements of resource allocation as a basis for using the matrix and determining severity of DDIs, these judgements need to be evaluated in relation to outcome measures such as hospital stay, morbidity, mortality, and resource use. A major weakness of the present matrix is that it requires manual work. Future development aims to simply its use and usability by, for instance, integrating a database query of DDIs and data from an expert panel to automatically highlight likely and relevant DDIs for a given MM cluster. A limitation at present is that all diagnoses in a database query of DDIs must be encoded or structured in a way that can be further processed by the web-based tool. This is not yet the case.

The main strength of the network graph is that it allows the user to visualize severity of interactions at one glance. Especially in a complex case, a simple visual overview of the pattern of DDIs might reduce the cognitive load of maintaining a mental representation of DDIs (see Fig. 1B).^[62]^ A further advantage is that the visual representation highlights the interactions according to the physician's own judgements of severity, making the network graph immediately relevant for the physician and the case. Potentially, the graph could augment the reporting of complex cases as a means to gaining a quick overview of the DDIs in a case and by highlighting potentially critical points of convergence between conditions and medications that require might particular attention. The graph cannot replace clinical contact to the patient. Empirical evaluation of the usability and effectiveness of the network graph for less and more experienced internists as a simple aid to representing, communicating and considering a complex case awaits investigation.

As a summary measure, the MISI may be used to gauge and communicate the general severity of a case. Importantly, the MISI provides a summary evaluation of the harmful interactions at a given point in time. As a momentary assessment, the value of the MISI will change over the course of treatment. With further development, the use of the MISI as a basis for measuring change in interaction severity over time will be tested. One priority for further development is to first ensure the reliability of this index. The index might then be used as a reference for typical MM clusters (and combinations of harmful DDIs), as established for example by an expert panel. The advantage of this is that a physician could judge his or her assessment of case severity against the reference. While the MISI is conceptualized as a comparative scale, empirically based thresholds of higher, medium, and lower risk are needed. These thresholds are likely to be time-consuming and costly to develop, but they would foster for purpose of research and clinical assessment consistent and meaningful interpretation within particular and across different MM clusters.

To consider potential strengths and limitations from the perspective of the user, we collected feedback from the physicians at debriefing. This supported the view that the matrix evoked greater reflection about and awareness of potential DDIs and therapeutic conflict. The network graph was not investigated, but the feedback supported the idea that the visualization could help to make the user more aware of these interactions and conflicts. On the other hand, the use of the matrix was reported by some as requiring a high level of attention. The task of explicitly judging the severity of DDIs generated a sense of uncertainty in some raters, in part because it revealed to the rater imperfect knowledge: internet links to an interaction checker were suggested. Clearly, the task was made more difficult in this study by the fact that anamnestic, clinical, and laboratory data were not provided.

**Figure 3:**
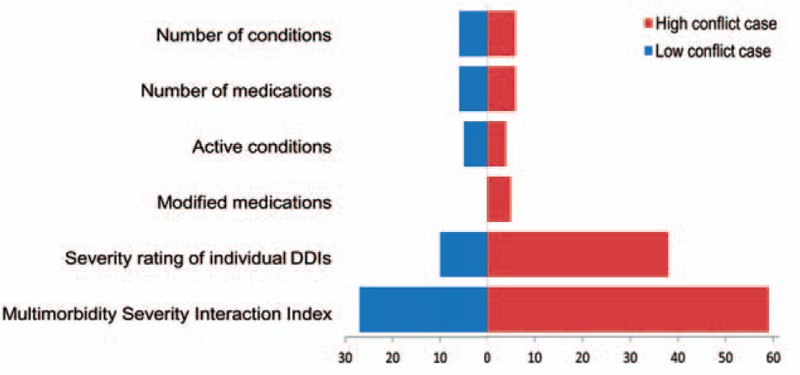
The figure illustrates the composition of the MISI, based on one physician's ratings of a low conflict case (right side of main panel) and a high conflict case (left side of main panel).

The data of this study show that quantifying the degree of MM simply on the basis of the cumulative number of concurrent conditions does not necessarily reflect the clinical effort needed to prioritize, plan, and manage harmful interactions. As indicated in Table 3 and Fig. 3, the total number of a patient's conditions and medications can deviate from the number of conditions and medications that the physician considered to be relevant for current treatment. While each patient case was designed to have 6 concurrent conditions, the analyses showed a significant difference between the low conflict and high conflict cases in terms of the judged need to modify the patient's medications; the high conflict case requiring a significantly greater number of modifications. The physician must also prioritize the clinical effort needed to manage and monitor more harmful interactions. The computation of the MISI considers, therefore, the cumulative number of concurrent conditions and medications, while weighing the index to take account of more severe DDIs that the physician considers to require more urgent attention. Further work is needed to ensure that the judgments of anticipated resource utilization and our weighting procedure reasonably reflect real resource utilization.

Other indicators of MM severity have been used.^[40]^ These range from composite measures based on multiple dimensions (e.g., prognostic threat to life, number of organs affected, disability, complications, and seriousness of treatment) to more simple uni-dimensional measures of severity.^[63,64]^ The MISI could be easily adapted to generate a composite score based on 1 or more other such dimensions. But the present definition of severity, in terms of resource utilization, serves to focus attention on the relationship between the use of health care resources and DDIs^[42]^ by considering the immediate relevance of the variously harmful interactions for clinical prioritization, planning, and management.^[65]^ This is important considering the impact of MM on health service utilization and costs.^[66–68]^ These costs increase exponentially with the increasing number of chronic diseases.^[69]^

## 5 Conclusion

The present study demonstrated that the MISI can be used as a measure of subjective judgement of harmful interactions to reliably distinguish between 2 hypothetic patients of potentially low and high therapeutic conflict. The judgement of harmful interaction is based on the internist's subjective assessment of the intensity of effort (i.e., resource utilization) required to clinically manage the harmful interactions. In conducting this proof of concept study, we made the assumption that the main source of systematic variation in the subjective judgements of resource allocation would be attributable to the differences in DDIs between the low and high conflict cases. But the impact of the internists’ expertise in treating MM and the validity of the subjective construct “resource utilization” itself needs to be subject to testing. Importantly, the MISI needs to be evaluated for a larger number of patients and for a greater range of variously severe MM. In supporting clinical decision making, the patient-specific MISI focuses attention on the patient as a whole, rather than on any 1 multimorbid condition, by highlighting the interacting conditions and treatments (cf.^[70]^). The visually intuitive network graph is designed to help the clinical decision-maker visualize his or her assessment of the case. The potential usability and effectiveness of the graph as an aid to representing, communicating, and considering a complex case awaits evaluation. The digital format of the MISI means that this instrument is practical to administer and easy to adapt for application in clinical practice and research.

## Acknowledgments

The authors thank Professor Beate Sick, Zurich University of Applied Science, Winterthur, Switzerland, for consultation regarding the statistical approach applied in this study.
